# Chloroform in Intermittent Fever

**Published:** 1856-04

**Authors:** N. Dalton

**Affiliations:** Logan, Ohio


*Chloroform in Intermittent Fever.* By N. DALTON, Logan, Ohio.

Some time in September last, I visited a young athletic man, laboring under an intermittent with general visceral congestion, which seemed to menace his life. I was convinced he must die unless he was speedily relieved. For promptitude, I was induced to try the internal use of Chloroform, and gave him about two drachms with a half gr. sulph. morphine. In a few seconds he fell asleep and slept soundly; his pulse, which could not be felt at the wrist, came up to about 90, full and soft, when he awoke, and to my astonishment expressed himself perfectly well. All the unpleasant symptoms had given way, nor was the cold stage followed by any feverish reaction. This all occurred in less than an hour. I was so much pleased with its effects that I concluded to test its anti-periodic properties by risking the probability of its return. From four to five weeks elapsed before it did return, during which time, and since, I have given it in the cold stage of quite a number of cases of simple intermittent, in doses varying from one to two drachms, in a little camphor water, both alone and connected with the morphine, and in every case have had the pleasure of witnessing the same prompt arrest of the disease, and in but two cases has there been any return of the ague, and in one only has there been any feverish reaction, but all are instantly arrested. I mentioned the matter to my partner, Dr. Hoffman, who has used the Chloroform in a number of cases with the same happy results; also to Dr. Pullen, who tried it on himself, with the effect of immediately arresting the chill, leaving him to feel as well as usual after an intermittent attack; but being fearful of its anti-periodic properties, took from two to three grains of quinine the next day, and has not had a return of the ague since.

I will report more fully as soon as I can get time, possibly during the coming month. In the meantime, I hope you will lay this before your readers, in some shape, that it may be more generally tried. If found to be as serviceable in the hands of others as in ours, it will be of incalculable benefit both in relieving human suffering and in a monetary point of view. Should it, on the other hand, serve no other purpose than so promptly to arrest those alarming and frequent fatal congestive chills, it will do much good.—*(Ohio) Medical and Surgical Journal.*

